# Atypical Hemolytic Uremic Syndrome Presenting as Acute Heart Failure—A Rare Presentation: Diagnosis Supported by Skin Biopsy

**DOI:** 10.1177/2324709619842905

**Published:** 2019-04-22

**Authors:** Asim Kichloo, Savneek Singh Chugh, Sanjeev Gupta, Jay Pandav, Praveen Chander

**Affiliations:** 1 CMU Medical Education Partners, Saginaw, MI, USA; 2 Westchester Medical Center, Valhalla, NY, USA

**Keywords:** microangiopathic hemolytic anemia, atypical hemolytic uremic syndrome, complement dysregulation, heart failure

## Abstract

Atypical hemolytic uremic syndrome (aHUS) is a rare disorder of uncontrolled complement activation that manifests classically as anemia, thrombocytopenia, and renal failure, although extrarenal manifestations are observed in 20% of the patient most of which involving central nervous system, with relatively rare involvement of the heart. In this article, we report the case of a 24-year-old male with no history of heart disease presenting with acute systolic heart failure along with microangiopathic hemolytic anemia, thrombocytopenia, and acute kidney injury. Given his presentation of thrombotic microangiopathy (TMA), along with laboratory results significant for low haptoglobin, platelets, hemoglobin, C3, C4, CH50, and normal ADAMTS13 levels, with no diarrhea and negative STEC polymerase chain reaction in stool, aHUS diagnosis was established with strong clinical suspicion, and immediate initiation of treatment was advised. Kidney biopsy to confirm diagnosis of aHUS was inadvisable because of thrombocytopenia, so the skin biopsy of a rash on his arm was done, which came to be consistent with thrombotic microangiopathy. Our case highlights a relatively rare association between aHUS and cardiac involvement, and the use of skin biopsy to support diagnosis of aHUS in patients who cannot undergo renal biopsy because of thrombocytopenia.

## Introduction

Hemolytic uremic syndrome (HUS) is characterized by nonimmune microangiopathic hemolytic anemia, thrombocytopenia, and acute kidney injury.^1^ Pathologically, HUS causes thickening of arterioles and capillaries, causing endothelial swelling and detachment in the target organ. This can lead to thrombosis and obstruction of microvessel lamina, which induces tissue ischemia.^2^ Although these lesions typically affect the renal vasculature, any organ including the brain, heart, lungs, liver, eyes, pancreas, and gastrointestinal tract may be involved.^1,2^ The majority of HUS cases are caused by Shiga toxin–producing *Escherichia coli* (STEC)^1^; however, some cases not involving Shiga toxin are labeled as atypical HUS (aHUS). Research over the last 20 years has shown that 60% of cases classified as aHUS are due to complement pathway dysregulation.^1^ Complement dysregulation occurs in the form of acquired or genetic mutations in genes encoding complement proteins. Antibodies to complement proteins have been implicated in the etiology of 6% to 10% of patients with complement-mediated HUS.^3-7^ Other cases of aHUS are termed secondary aHUS and are thought to be caused by drugs, pregnancy, malignant hypertension, nonenteric bacterial, and viral infections.^1^

Thrombotic thrombocytopenic purpura (TTP) is another subtype of thrombotic microangiopathy that is known to affect predominantly the brain and the heart.^8-10^ TTP is due to a severe deficiency in ADAMTS13 enzyme activity, a metalloprotease involved in the breakdown of Von Willebrand factor multimer, leading to widespread hyaline thrombosis affecting the small vessels.^3,11,12^ The cardiac injury manifested by this process is recognized as the leading cause of death in these patients.^8-10^ It is important to recognize that, albeit sparsely, cardiac events do affect patients of aHUS, leading to severe complications and death.^13-18^ Cardiac manifestations vary from myocardial infarction, cardiomyopathy, to acute decompensated heart failure.^3,10^ In a study done in 1997, 43% of children with aHUS (10 out of 23) developed heart failure that required inotropes. Two of those patients died from the aHUS-induced cardiomyopathy within 3 months.^4^ Recent studies have shown that aHUS patients with a genetic or acquired defect in CFH, a key regulator of the alternative complement pathway, are even more susceptible to developing such cardiac complications when compared with those aHUS patients without the acquired defect.^4,19^ The associated heart injury and dysfunction in these patients are mainly due to continuous activation of the complement system, leading to endothelial injury and thrombosis in the coronary microvessels.^7^

## Case Report

A 24-year-old male presented to the hospital with acute onset shortness of breath. Initial evaluation revealed cardiogenic shock, acute kidney injury (serum creatinine 2.54 mg/dL), and thrombocytopenia (platelet count 69 000). Heart catheterization revealed ejection fraction of 20%. Laboratory evaluation also revealed hematuria with red blood cell casts, proteinuria (0.7 g/dL), anemia (Hb 11.5 g/dL), low haptoglobin levels (<8), low C3, C4, and CH50 activity. ADAMTS 13 levels were normal (84% activity). There was no history of diarrhea, and STEC polymerase chain reaction (PCR) in stool was negative. ANA, p-ANCA, c-ANCA, hepatitis panel, and antiphospholipid Ab results were negative. Given the clinical picture, aHUS diagnosis was established and immediate initiation of treatment was advised. The patient also developed a skin rash on his arm during his hospital stay, which was biopsied. Histopathology showed features consistent with thrombotic microangiopathy with positive staining for fibrin and C4d confirming a diagnosis of complement-mediated microangiopathy or aHUS. He underwent spontaneous remission before complement blockade therapy could be initiated due to patient’s reluctance about the safety profile of immunotherapy medications and because of his wish of a second opinion. The patient was discharged after significant improvement of renal function, cardiac function, and normalization of platelet count, with a close follow-up at a higher level center.

## Discussion

aHUS is a rare disorder consisting of microangiopathic hemolytic anemia, thrombocytopenia, and multiorgan involvement.^1^ Unlike the more common STEC-HUS caused by Shiga toxin, aHUS is caused by uncontrolled overactivation of complement pathway due to genetic mutations or antibodies against complement regulatory proteins.^1^ aHUS causes about 10% of total cases of HUS and has an overall poor prognosis. The incidence rate of aHUS in the United States is about 2 per million.^20^ The kidney is the most commonly involved organ, although extrarenal manifestations are observed in 20% of the patient most of which involving the central nervous system, with relatively rare involvement of heart.^3,21,22^ Probable causes include high-output heart failure from anemia and microangiopathic injury in the coronary vasculature resulting in varying manifestations ranging from myocardial infarction, cardiomyopathy, to acute decompensated heart failure.^3,23^

**Figure 1.**
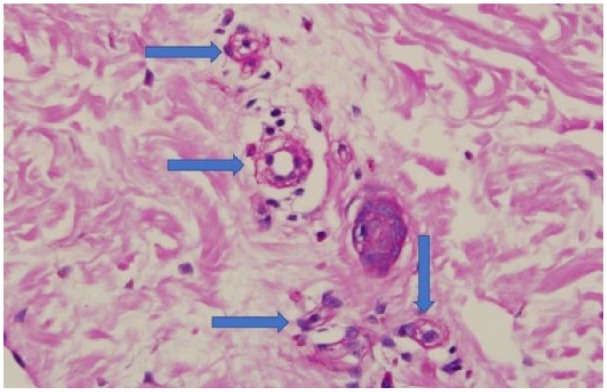
Representative PAS-stained image of skin biopsy showing several superficial arterioles; note the marked narrowing and obliteration of the lumen associated with edematous intimal thickening (depicted by blue arrows).

**Figure 2.**
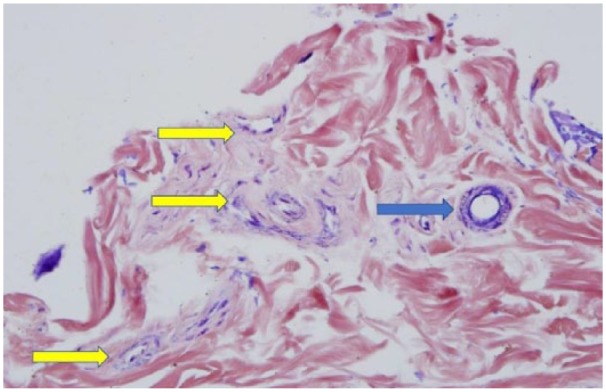
Positive staining for fibrin in one of the affected arterioles with PTAH stain (as shown by the blue arrow); note negative staining of the unaffected arterioles (as shown by the yellow arrows).

**Figure 3.**
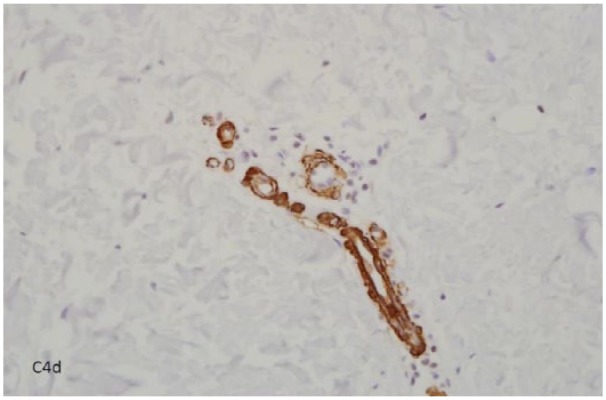
Strongly positive staining for C4d, a metabolic end product of complement pathway, in several arterioles showing edematous intimal thickening.

aHUS is diagnosed by the clinical picture of thrombotic microangiopathy with normal ADAMTS 13 activity (ADAMTS 13 activity >10%) to rule out TTP, and negative STEC PCR in stool to rule out thrombotic microangiopathy due to Shiga toxin.^24^ Our young patient had no previous history of heart disease and presented with acute shortness of breath. On cardiac catheterization, he was found to have acute systolic heart failure with ejection fraction of 20%. Our patient was also found to have hemolytic anemia, schistocytes on peripheral smear, thrombocytopenia, acute kidney injury, low haptoglobin levels, low C3, C4, CH-50 levels, and normal ADAMTS 13 levels, all of which suggestive of HUS. He did not report diarrhea and had a negative stool STEC PCR workup. In a clinical setting like this, performing a kidney biopsy was deemed inadvisable due to thrombocytopenia. However, since our patient also had a purpuric skin rash, a skin biopsy was done, which showed features consistent with thrombotic microangiopathy. His biopsy was also positive for staining with fibrin and C4d, which further supported the diagnosis (Figures 1-3).

Since most of these patients have concurrent thrombocytopenia and renal biopsy might be a challenge, a skin biopsy is much safer and may show specific changes of TMA and may be of significant value in corroborating a diagnosis of aHUS. Limited data are available on the role of skin biopsy in the diagnosis of aHUS as only single-center study not validated in multi central settings has shown the corroborative diagnostic potential of skin biopsy in a patient of aHUS.^25^

Treatment of aHUS includes complement pathway termination with eculizumab, a monoclonal antibody against C5, plasmapheresis, immunosuppression with steroids, rituximab, mycophenolate, and supportive therapy.^26^ In most of the reported case studies, patients who developed vascular stenosis also received hemodialysis as a part of their treatment. It is important to note that exposure of blood to the hemodialysis filter causes complement activation by generating C3a and C5b.^27^ This can potentially exacerbate the severity of the illness in patients with complement-related aHUS and lead to advanced vascular injury. This case emphasizes on aHUS with primary cardiac involvement, which also involved the kidney and where skin biopsy was used to support the diagnosis.

Eculizumab is a monoclonal antibody that binds to C5 and inhibits its breakdown to C5a and C5b and therefore prevents subsequent formation of the membrane attack complex (MAC).^28,29^ The feared complications of hemodialysis in complement-related aHUS can also be prevented by placing the patient on eculizumab during the treatment sessions.^30^ Since myocardial infarction and heart failure are life-threatening complications, early clinical suspicion leading to prompt diagnosis and initiation of therapy are of utmost importance to improve survival and long-term prognosis.
